# From yacht deck to climate lab: navigating currents and building climate resilience!

**DOI:** 10.3389/fphys.2026.1867608

**Published:** 2026-06-01

**Authors:** Jan G. Bourgois, Gil Bourgois, Toste Tanhua, Peter Landschützer

**Affiliations:** 1 Department of Movement and Sports Sciences, Faculty of Medicine and Health Sciences, Ghent University, Ghent, Belgium; 2 GEOMAR Helmholtz Centre for Ocean Research Kiel, Kiel, Germany; 3 Flanders Marine Institute (VLIZ), Ostend, Belgium

**Keywords:** climate change, human health, marine ecosystem, oceanography, resilience, sailing

## Abstract

Round-the-world ocean races such as *The Ocean Race* and the *Vendée Globe* expose sailors to some of the planet’s most remote and extreme marine environments, creating a unique opportunity to study both environmental conditions and human responses to them. In this comment we highlight how these events can function as mobile climate laboratories, enabling the simultaneous collection of oceanographic and atmospheric data alongside human biometric information, including physiological, psychological, and cognitive indicators. Integrating disciplines such as climatology, oceanography, ecology, and human health sciences enables investigation of how humans adapt to environmental stressors while also improving environmental monitoring. Establishing coordinated interdisciplinary research programs could strengthen climate resilience research, enhance ocean and human health monitoring, and support more sustainable engagement with marine environments.

## Introduction

On the edge between mastery and surrender, sailors in round-the-world ocean races embody the struggle of humanity itself – navigating uncertainty, volatility, and the relentless rhythm of a transforming sea. Covering 70% of the Earth’s surface and absorbing ~90% of excess heat and ~1/3 of annual anthropogenic carbon dioxide (CO_2_) emissions, the ocean is central to climate regulation but faces escalating acidification, warming, and marine heatwaves that threaten ecosystems, human health, and coastal livelihoods (Falkenberg and Dupont, 2023). Rising temperatures and climate variability disrupt Earth’s physical and biological homeostasis, driving more frequent and extreme events – heatwaves, droughts, wildfires, cold snaps, storms, and floods – with cascading impacts on environmental stability and human health. Given the unprecedented pace of these changes, enhancing resilience is a critical strategy to mitigate the catastrophic effects of climate change.

“Resilience is the art of navigating through torrents” (Cyrulnik, 2001). This metaphor resonates across disciplines, notably in oceanography and human health sciences. Resilience defines the capacity of systems to withstand disturbance, adapt to change, and recover function. In the ocean, it enables ecosystems to absorb stressors and maintain functionality (Zhai and Lee, 2024). In human health, it reflects a dynamic process of mobilizing resources, fostering physiological, psychological, and behavioral adaptability, and sustaining transformation (Jatobá, 2025). Strengthening resilience across marine and human domains is essential for long-term sustainability and global well-being.

**Figure 1:**
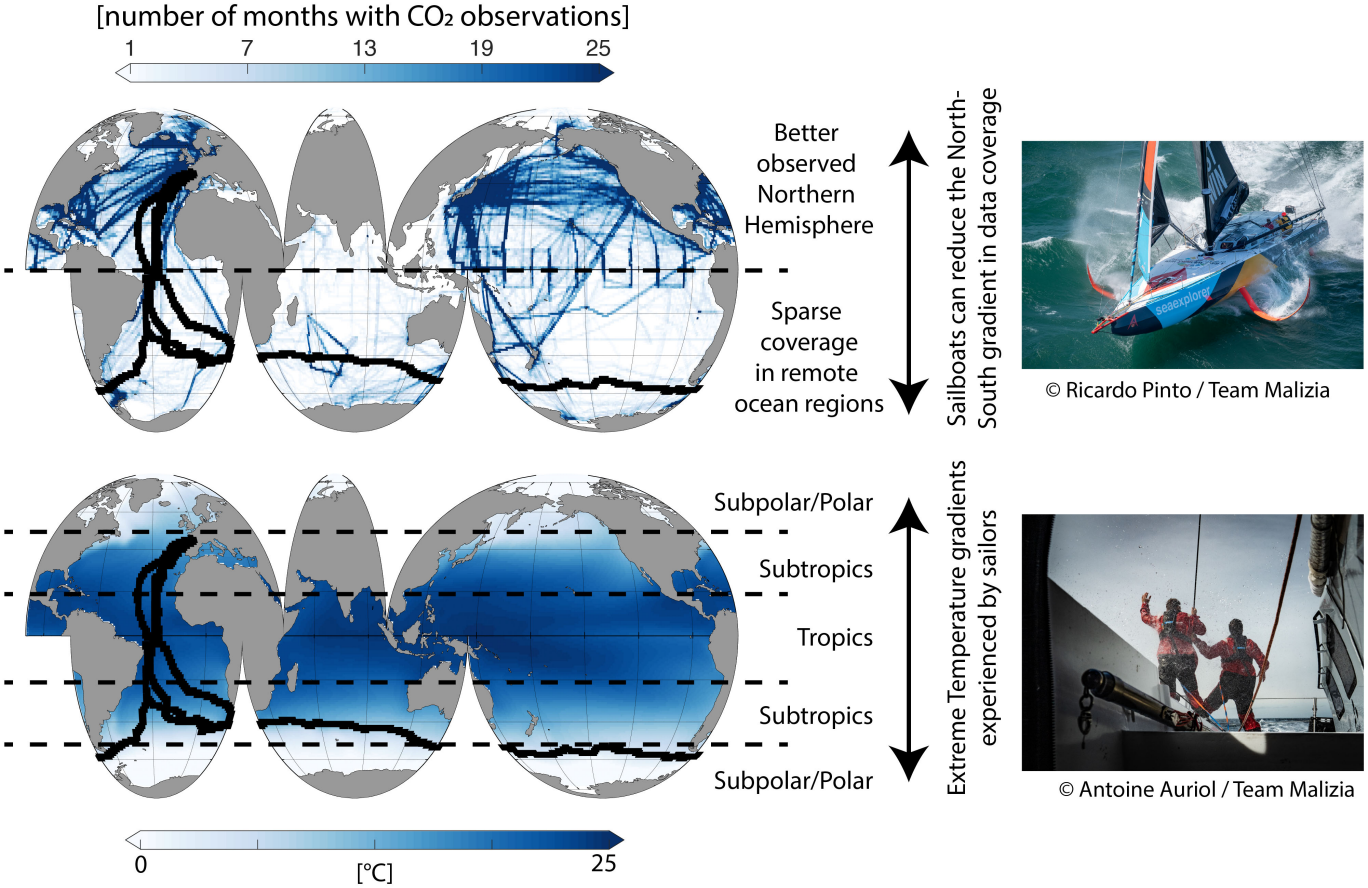
Illustration on how sailboat races (illustrated by black lines from the Vendée Globe Race in 2020/21 on the map) can help overcome data sparsity in remote ocean regions (based on Bakker et al., 2016, top), as well as help better understand the human response to extreme environmental variability illustrated by sea surface temperature variation (Rayner et al., 2003, bottom).

Professional round-the-world sailing races such as *The Ocean Race* (TOR; formerly *Whitbread Round the World Race* and *Volvo Ocean Race*, crewed, multi-leg, since 1973) and the *Vendée Globe* (VG; solo, non-stop, unassisted, since 1989) – reoccurring every 4 years – span some of the planet’s most remote and inhospitable marine regions, exposing participants to extreme environmental variability (**Figure 1**). Despite the considerable publicity surrounding both races and the implementation of scientific initiatives by organizing bodies and participants, few results are available to date through scholarly literature. The main reason for the sparseness of scientific literature is that sensors capable of making meaningful ocean and human health observations have only recently reached the required technical maturity to be deployed more regularly. Early studies, spanning the 1980s to the early 2000s, focused largely on technical and medical aspects of the sport. From the early 2000s onward, research adopted a more holistic approach, highlighting interactions between the sport and environmental factors. Beyond their technological and medical relevance, endurance ocean-racing vessels provide a unique platform for interdisciplinary research.

The ocean has been referred to as “Earth’s inner space” because it remains largely underexplored, with many new discoveries to be made, similar to outer space. The ocean and space relationship is close, since it is not possible to understand the ocean without information from space observations: satellite-based data on temperature (e.g. Rayner et al., 2003) and other variables are essential for mapping the global CO_2_ flux. Furthermore, the ocean and space are also similar in that both environments pose significant challenges for technological systems as well as the human body.

### Ocean systems under stress: monitoring marine resilience in motion

One of the core climate services provided by the global ocean is its ability to absorb CO_2_ at the air-sea interface and store it in the deep for decades to centuries, however, at the cost of the ocean acidification (Intergovernmental Panel On Climate Change (Ipcc), 2023). The key driver of CO_2_ uptake at the air-sea interface is the concentration or partial pressure difference at the air-sea interface. While CO_2_ in the surface atmosphere is well mixed and can be well constrained through a relatively small number of measurement stations, this is not the case for CO_2_ dissolved in the surface ocean. Here, *in-situ* measurements are required to capture the temporal and spatial scales needed to infer variability imposed through climate change. Our present-day observing system, however, contains large data gaps in the South Atlantic, the Southeast Pacific and Southern Ocean (Bakker et al., 2016), i.e., regions that are an integral part of the reoccurring TOR and VG tracks. To a large extent, these measurement gaps are the result of the remoteness of these ocean basins, the rough conditions ships encounter and the generally lower ship traffic compared to the Northern Hemisphere. It is thus evident that sailboat races have the potential to fill known data gaps and enhance fundamental climate science at its core. To date, however, few studies exist, building on physical (temperature, salinity) (Hernani et al., 2025), chemical (CO_2_) (Landschützer et al., 2023), or pollution (plastic) (Tanhua et al., 2020) measurements. Additionally, none of them have investigated compound effect, despite the literature having highlighted the added value of these rare measurements to quantify the marine CO_2_ uptake (Behncke et al., 2024; Behncke et al., 2026), particularly in the Southern Ocean, i.e., the largest marine carbon sink on our planet. The potential of the synergy between science and sailing, however, goes far beyond present-day studies and current measurement set-ups. Climate change poses an ongoing threat to the global oceans leading to stress to marine ecosystems and human society. Continuous monitoring the fate of CO_2_ and ecosystem health is thus a necessary lens into our future. Given the reoccurring nature of these races, the large sample size from multiple boats taking part in the races and the geographic diversity, the TOR and VG provide the ideal platform to monitor long-term climate impacts, progressing ocean acidification but also marine pollution and ecosystem changes. Continuous monitoring efforts may provide a time series at different climatic regions, from the tropics to the high latitudes and if coordinated well, they may provide unprecedented knowledge on progressing climate change and ecosystem health, allowing us to obtain an unequivocal constraint on ecosystem resilience in response to marine stressors.

### Human health under stress: monitoring human resilience at sea

TOR and VG constitute some of the most demanding multidisciplinary endurance challenges, conducted on high-performance vessels in harsh, variable conditions. With minimal onboard comfort and limited access to external support, these races place exceptional demands on human resilience. Sustained performance in these settings requires a complex interplay of physiological, psychological, and cognitive capacities to mitigate fatigue and maintain health by counteracting injuries, illnesses, and other survival-related stressors. The acquisition and analysis of physiological, psychological and cognitive data from sailors exposed to compounded internal (e.g., sustained physical exertion, circadian disruption and thermoregulatory strain) and external (e.g., sea state, diurnal variability and meteorological extremes) stressors, is increasingly pertinent. To date, few studies (TOR: 6 publications; VG: 2 publications) have systematically assessed sailors’ health and performance during offshore sailing. Existing studies have primarily focused on physiological and health-related outcomes, including injuries and illnesses (Leach, 1978; Bugge, 1986), anthropometrics and body composition (Branth et al., 1996; Branth et al., 2007; Fearnley et al., 2012; Philippe et al., 2022), energy expenditure (Bugge, 1986), and dietary and fluid intake (Branth et al., 1996; Branth et al., 2007; Fearnley et al., 2012; Philippe et al., 2022; Komarzynski et al., 2024). Other research has examined performance-related factors such as physical activity and training characteristics (Branth et al., 2007; Philippe et al., 2022; Komarzynski et al., 2024), while a smaller body of literature has investigated psychological and recovery-related aspects, including sleep (Komarzynski et al., 2024), and stress and recovery (Gunnarsson et al., 2004; Philippe et al., 2022; Komarzynski et al., 2024). However, most of these studies were limited to specific segments of the races – lasting days, weeks, or an individual stage – rather than encompassing their full duration. Thermoregulation has been largely overlooked, despite its potential implications for sailors’ health, safety and performance. Human thermoregulatory responses are determined by the balance between metabolic and environmental heat gain and heat dissipation, which in turn depends on the interaction between ambient weather conditions (i.e., temperature, humidity, radiation and wind) and individual characteristics (e.g., age, sex, ethnicity, anthropometrics, body composition, health status, fitness level, sleep deprivation, nutritional and hydration status). Prolonged exposure to heat, cold, or cold water poses significant challenges to the stability of core body temperature and interacts with fundamental determinants of human functioning and performance. Rapid changes in weather and sea conditions appear to limit the efficacy of acclimatization, habituation, and cross-acclimatization strategies employed in preparation for, and during TOR and VG. Consequently, behavioral strategies, such as deliberate activity pacing, informed clothing selection, and management of microclimates within the vessel, are likely to play a pivotal role in mitigating thermal stress. In this perspective, integration of heat-flux monitoring devices as body-worn sensors or clothing-integrated systems could provide valuable insights into thermoregulation and the thermal strain (due to both heat and cold stress) experienced by sailors during extreme offshore races. Emerging technologies also show promise for non-invasive hydration monitoring, for example through wearable-integrated biosensing solutions. In addition, commercially available wearables offer opportunities to continuously monitor cardiovascular load (heart rate), autonomic nervous system function (heart rate variability; HRV), sleep quality and quantity, stress and recovery status, and other health metrics. Furthermore, collection of saliva samples with robust portable point-of-care devices could provide additional information on biomarkers related to physiological stress (e.g., cortisol) and immune function (e.g., immunoglobulin A). Together, these measurements could contribute to a comprehensive understanding of how sailors are affected by sustained physical exertion, sleep deprivation and prolonged exposure to extreme environments during remote sailing races. Generating robust evidence in this field is not only critical for sailors’ health, safety and performance, but may also inform broader strategies for climate resilience, occupational safety in increasingly unpredictable environments, and coping with physiological, psychological and cognitive pressures faced by modern society.

## Conclusions and recommendations

TOR and VG traverse some of the planet’s most remote and inhospitable marine regions and exposing participants to a wide spectrum of extreme environmental conditions and stressors. It is therefore a very welcome development that both races require participants to further participate in some form of ocean observing during the races, however usually focusing on the environmental component. Functioning as mobile climate laboratories, they can facilitate the simultaneous acquisition of environmental data, such as oceanographic and atmospheric parameters, and human biometric information encompassing physiological, psychological, and cognitive responses and adaptations. Despite logistical challenges, this dual exposure supports integrated studies at the intersection of environmental science and human health. Integrating climatology, oceanography, ecology, and human health sciences support the development of adaptive strategies and strengthens the abilities for surveillance, foresight, and knowledge acquisition that sustain ongoing improvement. These processes are essential for advancing climate resilience, safeguarding ocean and human health, and promoting sustainable interaction with marine environments. Research in extreme environments occurs outside controlled laboratory settings, imposing constraints on technologies for capturing both oceanographic and human biometric data. Studies during TOR and VG therefore demand monitoring systems that are innovative, robust, and methodologically simple. Previous work has shown that novel sensor technology, that allows for automated sampling for several weeks to not burden the sailors on their main endeavor of completing the physically most challenging sailing events, is feasible to combine professional racing with climate data collection (Landschützer et al., 2023). Moreover, the continuous monitoring of physiological, psychological and cognitive parameters through biosensors and biomarkers, alongside satellite-, web-, and mobile-based platforms, has advanced substantially over the past decade and is anticipated to expand exponentially in the coming years. Within this context, TOR and VG could pioneer research on human performance, climate resilience, and ocean and human health combined, while integrating emerging technologies to advance applied environmental and health sciences. Ethical and practical challenges in monitoring during offshore sailing races require close collaboration between researchers, race organizers, boat owners, and sailors. Clear informed consent and data governance agreements are essential to define data use, ownership, privacy, and participant rights. Technical limitations, including sensor malfunction or data loss in extreme environments, must also be considered, particularly in non-stop races such as VG. To address these challenges, TOR and VG organizers together with academic partners should establish a well-structured and robust interdisciplinary scientific program, developed in consultation with all relevant stakeholders and modelled on established approaches used in space exploration.

## Data Availability

The original contributions presented in the study are included in the article/supplementary material. Further inquiries can be directed to the corresponding author/s.
